# Effects of Different Fermentation Methods on Flavor Quality of Liupao Tea Using GC-Q-TOF-MS and Electronic Nose Analyses

**DOI:** 10.3390/foods13162595

**Published:** 2024-08-19

**Authors:** Xiaohui Zhou, Di Tian, Hongjie Zhou, Rui Dong, Chenyang Ma, Ling Ren, Xueyi Yang, Qingyi Wang, Ning Chen, Liubo Yang, Xuan Tang, Yixin Bi, Yapeng Liu, Xiujuan Deng, Baijuan Wang, Yali Li

**Affiliations:** 1 College of Tea Science, Yunnan Agricultural University, Kunming 650500, China; 18887449386@163.com (X.Z.); 1984020@ynau.edu.cn (H.Z.); 1720678821@163.com (R.D.); machenyang0305@icloud.com (C.M.); 15750317329@163.com (L.R.); yangxueyi202202@126.com (X.Y.); 18881117477@163.com (Q.W.); cn505879143@163.com (N.C.); y1578280950@163.com (L.Y.); 19988155297@163.com (X.T.); 18287775024@163.com (Y.B.); a13180512869@163.com (Y.L.); 15808869561@163.com (X.D.); 2 College of Food Science and Technology, Yunnan Agricultural University, Kunming 650500, China; m17853435722@163.com; 3 Yunnan Organic Tea Industry Intelligent Engineering Research Center, Yunnan Agricultural University, Kunming 650201, China

**Keywords:** Liupao tea fermentation, *Monascus purpureus* inoculation, GC-Q-TOF-MS, electronic nose, sensory evaluations, odor activity value

## Abstract

To further develop Liupao tea products and enhance their flavor, this study investigated the effects of different fermentation methods on the aroma quality of Liupao tea. The aroma quality of Liupao tea was comprehensively analyzed using HS-SPME in combination with GC-Q-TOF-MS, electronic nose, and sensory evaluations. Electronic nose detection showed that the aroma fingerprints of Liupao tea samples with different fermentation methods were different. Sulfides, alcohols, ketones, and methyls were the main aroma categories affecting the aroma of the four groups of Liupao tea samples. GC-Q-TOF-MS analysis revealed significant differences in the composition of aroma components among the four fermentation methods of Liupao tea (*p* < 0.05). Furthermore, the total amount of aroma compounds was found to be highest in the group subjected to hot fermentation combined with the inoculation of *Monascus purpureus* (DMl group). Based on the OPLS-DA model, candidate differential aroma components with VIP > 1 were identified, and characteristic aroma compounds were selected based on OAV > 10. The key characteristic aroma compounds shared by the four groups of samples were 1,2,3-Trimethoxybenzene with a stale aroma and nonanal with floral and fruity aromas. The best sensory evaluation results were obtained for the DMl group, and its key characteristic aroma compounds mainly included 1,2,3-Trimethoxybenzene, nonanal, and cedrol. The results of this study can guide the development of Liupao tea products and process optimization.

## 1. Introduction

Liupao tea is a type of dark tea, and the finished product has unique quality characteristics, including a black and brown oily appearance, a red and thick beverage color, a mellow taste, betel nut and stale aromas, a brown leaf base, and resistance to brewing [1]. In recent years, it has gained popularity among consumers due to its health benefits, such as lowering blood lipids [2], improving obesity [3], and regulating intestinal flora [4].

The traditional processing techniques of Liupao tea include cold fermentation and hot fermentation, both of which are crucial for developing the distinct qualities of the tea. The hot fermentation process, also referred to as the “double steaming and double pressure” method, includes initial steaming, pile fermentation, re-steaming, drying or pressing, and aging [5]. In contrast, cold fermentation does not require the initial steaming step before fermentation. Comparing the two techniques, the advantages of hot fermentation are that it occupies the site for a short period of time and is more efficient. However, the degree of transformation of the raw material achieved within this short period of time is not significant, and a longer aging period may be required to obtain the desired red and thick quality. On the other hand, the advantage of cold fermentation is that it is energy-efficient and allows for better control of the degree of fermentation. However, it requires a longer fermentation time and occupies a long period of time at the site [6]. Microorganisms play an important role in the fermentation process of Liupao tea. However, traditional production methods face challenges such as long fermentation cycles, difficulty controlling fermentation strains, and inconsistent product quality [7]. To address these issues, it is essential to continuously optimize and improve the traditional Liupao tea fermentation process, develop new processing equipment, control the microbial species during fermentation, stabilize product quality, and advance the research and development of high-quality Liupao tea products.

In recent years, there has been a growing interest in innovative fermentation methods aimed at enhancing the quality of Liupao tea. Zhang [8] conducted a study to identify the most suitable new process combination for Liupao tea by investigating the impact of exogenous enzymes and fermentation conditions on the fermentation rate and quality of Liupao tea. Yang [9], in addition, carried out an artificial inoculation fermentation test on Liupao tea. By combining five dominant strains with seven different treatments, Yang found that the addition of exogenous microflora could lead to significant transformations in the composition of Liupao tea. Wu et al. [7] fermented Liupao tea with the inoculation of “*E. cristatum*”, resulting in a notable enhancement in the quality of Liupao tea. Similarly, Long et al. [10] used high-throughput sequencing to analyze the microbial community composition of Liupao tea at different stages of can fermentation, which provided a basis for innovating Liupao tea fermentation technology and leveraging microbial strains for improved outcomes.

As one of the microorganisms with a long history of applications in food processing in China, *M. purpureus* is now widely employed in the field of food fermentation [11,12,13]. In addition, *M. purpureus* has been used several times in the fermentation of post-fermented tea. Deng et al. applied a strain of *M. purpureus* (independent patent, 201010182965.9) capable of producing lovastatin to ferment Pu-erh tea, resulting in Pu-erh tea with a distinctive “Chenquan” aroma, and extensively examined its characteristic aroma [14]. Tian et al. [15] revealed the specificity of aroma in Pu-erh tea through different fermentation methods of *M. purpureus*. However, no relevant studies have investigated the flavor quality of Liupao tea fermented by *M. purpureus*. This study aims to comprehensively investigate the effects of different fermentation methods under the action of *M. purpureus* on the characteristic aroma of Liupao tea.

The electronic nose (E-nose) is an olfactory system that simulates the mammalian nose. It utilizes a sensor array to detect the aroma of samples, allowing for direct access to the overall flavor characteristics. In many cases, E-noses are combined with gas chromatography–mass spectrometry for aroma detection, aiding in quality identification, grading, and traceability of food products [16,17]. Rusinek et al. used an electronic nose to analyze the aroma intensity of Arabica coffee beans with three roasting degrees, and determined the intensity of coffee blend aroma in real time, which is of great significance for objectively evaluating the aroma intensity changes during coffee roasting [18]. Currently, the most commonly used technique for extracting and detecting tea aroma components is headspace solid phase microextraction–gas chromatography–mass spectrometry (HS-SPME-GC-MS) [19,20]. Compared to gas chromatography–mass spectrometry (GC-MS), GC-Q-TOF/MS offers the advantage of high-resolution time-of-flight mass spectrometry. This allows for the accurate determination of the relative molecular mass of compounds, coupled with the high separation ability of gas chromatography. As a result, GC-Q-TOF/MS can efficiently, rapidly, and accurately detect the aroma of samples. This technique is particularly useful for extracting target compounds in complex systems, thereby reducing the risk of misdetection and omitting detections [21,22]. The GC-Q-TOF/MS technique has also been increasingly utilized for the detection of tea aromas [23,24]. In this study, the aroma of four types of Liupao tea samples was analyzed using GC-Q/TOF-MS and an E-nose. The findings aim to establish a theoretical framework for regulating the flavor of Liupao tea, fostering innovation and product development, which is conducive to enhancing consumers’ perception of Liupao tea.

## 2. Materials and Methods

### 2.1. Preparation of Tea Samples

The Guangxi local baked green tea, made from *Csinensis var. pubil imba cv. Longsheng-longjicha*, was used as the raw material for the study. The tea samples were wetted with either 30% water or *M. purpureus* liquid. The *M. purpureus* liquid, which accounted for 5% of the weight of the tea sample, was mixed with water, which accounted for 25% of the weight of the tea sample. The tea samples underwent fermentation in a chamber with an average temperature of 25 °C and a humidity of 75%. The tea heap was turned over every 6 d, and water or *M. purpureus* liquid was added to maintain suitable temperature and humidity levels for uniform fermentation. On the 35th day of fermentation, samples were collected using the 5-point sampling method, with technical replicates, and then mixed and aliquoted into three equal portions for analysis. In the experiment, Cl represents the finished product of Liupao tea processed by cold fermentation, while Dl represents the finished product of Liupao tea processed by hot fermentation. CMl and DMl represent the finished products of Liupao tea processed by cold and hot fermentation, respectively, combined with *M. purpureus* inoculation. Specific details regarding the processing of “the *Monascus purpureus* strain” (independent patent, 201010182965.9) used in the experiments are provided in Supplementary Figures S1 and S2.

### 2.2. Experimental Equipment

The PEN3 E-nose was purchased from Airsense (Schwerin, Germany). Ethyl decanoate (99.99% purity for quantitative analysis) was purchased from Sigma-Aldrich LLC, Shanghai, China. N-hexane (chromatographically pure) was purchased from Merk (Merk KGaA, Darmstadt, Germany). NaCl was obtained from China National Pharmaceutical Group Corporation. C7-C40 (chromatographically pure) saturated alkanes and standards were purchased from Sigma-Aldrich (Darmstadt, Germany).

### 2.3. Sensory Evaluation

#### 2.3.1. Traditional Sensory Evaluation

According to the national standard GB/T 23776-2018 “Tea Sensory Evaluation Method”, seven professional tea reviewers (three males and four females, aged 24–61 years) from Yunnan Agricultural University used a combination of comments and scores to comprehensively evaluate the quality characteristics of Liupao tea samples [25]. Three grams of the tea sample was weighed and placed into a special cup for evaluation. After soaking in 150 mL of boiling water for 2 min, the tea infusion was leached out and the preliminary sensory evaluation was conducted. A second brewing was then carried out for 5 min, and the tea infusion was leached out again for further sensory evaluation. The final sensory evaluation results were obtained by combining the two sensory evaluations.

#### 2.3.2. Quantitative Descriptive Analysis

As a commonly used method for evaluating the characteristic attributes of samples, quantitative descriptive analysis (QDA) can provide a more accurate and intuitive description of the sensory attributes of the samples, making it a comprehensive sensory method capable of quantifying the results of sensory evaluation. This method has been widely used in the sensory evaluation of various types of tea samples [26]. In order to conduct a more comprehensive and scientific analysis of the sample aroma, the QDA method was used to further evaluate the specific aroma characteristics of the samples, building on traditional sensory reviews. The methodology followed by Mardiana et al. [27] was referenced for this purpose. The reviewers categorized the aroma descriptors of each sample based on the sensory evaluation results and identified five key quality descriptors to best represent the aroma qualities of the four samples: floral, fruity, herbal, stale, and woody. The intensity of each sample’s characteristic aroma was evaluated on a scale ranging from 0 to 6 (0 = no sensation, 1 = very weak, 2 = weak, 3 = slightly weak, 4 = slightly strong, 5 = strong, and 6 = very strong). Three sensory evaluations were carried out, and the intensity values of the characteristic aroma attributes were averaged to obtain the final QDA results.

### 2.4. E-Nose Analysis

Before the test, the E-nose system was preheated, and once it reached the working temperature, the airway was cleaned with filtered air to calibrate each sensor to the baseline. Subsequently, the direct headspace injection method was used for the analysis. First, 1 g of the tea sample was carefully weighed and placed in a 100 mL conical flask, ensuring an even distribution at the bottom. The flask was then sealed with a double-layer sealing film and allowed to stand at room temperature for 30 min before testing. The sampling interval was set at 1 s, with cleaning taking 60 s, zero adjustment 10 s, pre-sampling 5 s, and testing 120 s, and the injection volume flow rate was set at 300 mL/min. Three replicates of each sample were weighed and three parallel tests were conducted. The average response values obtained between 78 and 80 s were used for data analysis [28]. A comprehensive overview of each sensor in the E-nose system is provided in Table S1.

### 2.5. Extraction and Analysis of Volatile Compounds

#### 2.5.1. Pre-Treatment of Tea Samples

The test samples were ground into tea powder, sieved through a 40-mesh sieve, and approximately 10 g of tea powder was prepared for each sample. The prepared samples were then placed in tinfoil bags and stored in a refrigerator at −80 °C for GC-Q-TOF-MS detection.

#### 2.5.2. Headspace Solid-Phase Microextraction of Volatiles

The samples were removed from the freezer at −80 °C. Next, 0.5000 g of tea powder was accurately weighed and transferred to a 20 mL headspace vial; 20 µL of ethyl caprate internal standard solution was added, and the samples were equilibrated in an oven at 80 °C for 10 min. HS-SPME extraction was performed at a constant temperature of 80 °C by inserting a 120 µm DVB/CAR/PDMS extraction head into the headspace vial containing the samples. After 1 h of headspace extraction, the sample was resolved at 250 °C for 5 min, followed by GC-MS analysis. It is important to note that the extraction head was heated and aged in a Fiber Conditioning Station for 30 min before sampling.

#### 2.5.3. Conditions for GC-Q-TOF-MS Analysis

Chromatographic conditions: high-purity helium carrier gas (with purity no less than 99.999%) at a constant flow rate of 1.0 mL/min; DB-5MS capillary column (30 m × 0.25 mm, 0.25 μm); the inlet temperature was 250 °C, with split-less injection. Temperature programming: the initial temperature was 40 °C, and this temperature was maintained for 3.5 min, heating at a rate of 10 °C/min until the temperature rose to 100 °C, then heating to 180 °C continued at a rate of 7 °C/min, and finally heating to 280 °C occurred at a rate of 25 °C/min, and this temperature was maintained for 5 min.

The mass spectrometry conditions were as follows: electron bombardment (EI) ion source; electron energy: 70 eV; ion source temperature 230 °C; ionization mode, electron bombardment mode (EI); data acquisition method, TOF-Scan full scan; the scanning mass range was *m*/*z* 50~550, and the acquisition rate was 5 spectra. The quadrupole temperature was 150 °C, and the mass spectrometry interface temperature was 280 °C. The electron energy was 70 eV and the solvent delay was 3.5 min.

### 2.6. Qualitative and Quantitative Analysis of Volatiles

Qualitative Analysis Workflows B.08.00 software was used for analysis. After the analysis was completed, the original data were downloaded, and the total ion flow diagram obtained was used for qualitative analysis. Mass Hunter quantitative Analysis software B.06.00was then used to access the sample and download the mass spectrometry file. Subsequently, the reference values from the NIST online database (https://webbook.nist.gov/chemistry (accessed on 18 April 2024)) were matched, and the retention index and CAS number were used for qualitative analysis as outlined by Nie et al. [29]. The relative quantification of volatile components was calculated according to the method established by Fang et al. [30], with the unit of µg/kg. The formula for calculating the relative content (µg/kg) of volatile components is as follows:

Content (µg/kg) = target volatile component peak area × internal standard mass (µg)/internal standard peak area × tea mass (kg)

### 2.7. OAV Calculation for Volatiles

Based on the quantification of various volatile components, the odor activity value (OAV) of each component was calculated by the ratio of the relative content of the volatile substance (µg/kg) to the aroma threshold of the substance in water (µg/kg) according to the flavor threshold (OT) of each volatile substance in water based on the references. Typically, an OAV ≥ 1 for a volatile aroma component is regarded as the key aroma component that contributes significantly to the overall aroma of the sample [31].

### 2.8. Data Processing

The data from various samples were analyzed and categorized using Microsoft Excel 2016. Area stacked plots, histograms, Sankey plots, and radar plots were generated using Origin 2022 software v9.900225. Thermograms and Wayne plots were created using TB-tools software v2.119. Additionally, principal component analysis (PCA) was performed using a software cloud platform. Hierarchical cluster analysis (HCA) and orthogonal partial least squares discriminant analysis (OPLS-DA) were performed using SIMCA 14.1 software (V16.0.2, Sartorius Stedim Data Analytics AB, Umea, Sweden). An independent samples *t*-test was performed with SPSS 26.0 to determine the level of significant difference. Variable importance in projection (VIP) scores was obtained according to OPLS-DA, combined with an independent samples *t*-test at *p* < 0.05. Screening for differential aroma substances was conducted with VIP > 1, *p* < 0.05, and three measurements were obtained in parallel for each sample.

## 3. Results and Discussion

### 3.1. Results of Sensory Evaluation

#### 3.1.1. Results of Traditional Sensory Evaluation

The sensory evaluation of tea plays a crucial role in scientifically assessing sample quality and guiding tea production [32]. The evaluation factors of the four groups of samples were comprehensively assessed, as shown in Table 1. Among the four groups, in terms of appearance, the samples of the Cl and CMl groups, which underwent a cold fermentation process, exhibited a tighter and firmer appearance, while the samples of the Dl and DMl groups, which underwent a hot fermentation process, appeared looser. The color of the appearance and leaf base varied among the four groups, with the Cl and Dl groups, which were not inoculated with *M. purpureus*, displaying a brownish color, in contrast to the CMl and DMl groups, which were inoculated with *M. purpureus*, showing a reddish-brown color. Studies have shown that Aspergillus can produce a variety of enzymes closely related to the theabrownin biotransformation during dark tea fermentation, such as glycosyltransferase, glycoside hydrolase, and tannase. These enzymes effectively catalyze the hydrolysis, transformation, oxidation, and biodegradation of tea polyphenols, leading to an increase in theabrownin content and a deepening of the dark tea color. This finding is consistent with the results of this study [33]. In terms of taste, the Cl and Dl groups were described as rich and full-bodied with a sweet aftertaste, while the CMl and DMl groups, which were inoculated with *M. purpureus*, exhibited a sweet and mellow taste with a sweet aftertaste, albeit CMl displayed a thicker and smoother taste. In terms of aroma, each of the four groups of samples possessed distinct characteristics, with the exception of an overall stale essence. Specifically, the Cl group displayed a stale aroma with medicinal notes, the CMl group had a robust woody, stale, and fruity aroma, the Dl group featured a stale, floral, and fruity aroma, and the DMl group exhibited a strong stale aroma along with woody, flowery, and fruity notes, all of which were lasting. The appearance, infusion color, aroma, taste, and leaf base were scored comprehensively, with the DMl group receiving the highest score and being deemed the best in quality. These results indicate that the inoculation and fermentation of *M. purpureus* were more conducive to producing the reddish-brown color of Liupao tea, while also enhancing taste and aroma. Samples inoculated and fermented by *M. purpureus* exhibited a sweeter and mellow taste, intensified aroma, and enriched aroma varieties. The results are consistent with those obtained by Tian et al. [15].

#### 3.1.2. Quantitative Descriptive Analysis Results

**Figure 1:**
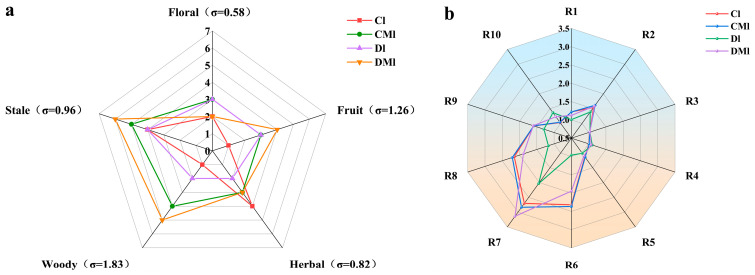
Aroma profile of different Liupao teas by QDA. (**a**) Electronic nose odor fingerprints of different Liupao tea (**b**).

In QDA, the magnitude of the sum of the aroma characteristic intensity values can indicate the advantages and disadvantages of the aroma of a group of samples to a certain extent. Meanwhile, the standard deviation of the aroma intensity values can assess the flavor stability of tea, with a negative correlation between the standard deviation and flavor stability [34]. The QDA results indicated that all four groups of samples exhibited a stale aroma, yet they displayed differences in the intensity of aroma characteristics, as shown in Figure 1a. The Cl group exhibited a strong herbal aroma compared to the CMl, Dl, and DMl groups; the Dl group had a strong stale aroma and weak floral and herbal aromas; the CMl group displayed a strong stale and woody aroma; and the DMl group exhibited strong stale, woody, and fruity aromas. The sum of the characteristic aroma intensity values of the four groups of samples ranked as follows: DMl (20) > CMl (18) > Dl (14) > Cl (12), indicating that the DMl group had the most favorable overall aroma profile. The standard deviation of aroma characteristic intensity values ranked as “woody” (1.83) > “fruity” (1.26) > “stale” (0.96) > “herbal” (0.82) > “floral” (0.58), suggesting that the aroma stability of “woody” was low, while “floral” aroma was the most stable. In general, the DMl group outperformed the other three groups in terms of the “stale”, “woody”, and “fruity” aroma attributes, suggesting that hot fermentation combined with *M. purpureus* inoculation was more conducive to producing a variety of aromas. Although sensory evaluation can assess the overall aroma characteristics of the samples, further analysis of specific key aroma components using instrumental testing is warranted.

### 3.2. Results of E-Nose

In order to gain a better understanding of the differences in the response signals of Liupao tea, processed using various fermentation methods, to different odors, a radar map was created using the average stable values of the response signals of 10 sensors (Figure 1b). The results indicated that the odor fingerprints of Liupao tea varied depending on the processing method employed. Specifically, sensors R2, R6, R7, and R8 showed higher response values. Additionally, the response values differed among Liupao tea samples processed with different techniques, with sensors R6, R7, and R8 exhibiting significant differences. Conversely, sensors R1, R2, R3, R4, R5, R9, and R10 demonstrated minimal variations, indicating that these sensors were not sensitive to the odor of the four Liupao tea samples. Overall, all four sample groups exhibited peak response values at sensor R7, indicating a prominent sulfide odor in the samples. A previous study on the modeling of the “stale” aroma of Liupao tea using an E-nose found that sensors W1S (methyls), W1W (inorganic sulfides), and W2S (alcohols, ketones) contributed more to the response of aged Liupao tea, which is consistent with the results of this study [35].

### 3.3. Analysis of Volatile Aroma Components

**Figure 2:**
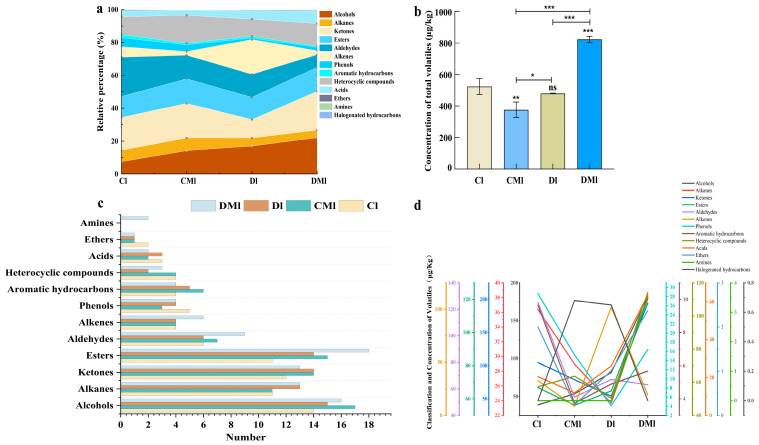
Proportion of volatile components in four types of tea samples (**a**) Histogram of the total concentration of volatile compounds of four samples (ns: *p* > 0.05, * *p* < 0.05, **: *p* < 0.01, ***: *p* < 0.001) (**b**). Volatile aroma species of four groups samples (**c**). Four groups of samples of different types of aroma content line chart (**d**).

As presented in Table S2, a total of 156 volatile aroma substances belonging to 13 categories were identified in the four groups of aroma samples. These included 26 alcohols, 26 alkanes, 25 esters, 24 ketones, 15 aldehydes, 10 alkenes, 9 phenols, 7 aromatic hydrocarbons, 5 heterocyclic compounds, 4 acids, 2 ethers, 2 amines, and 1 halogenated hydrocarbon. The aroma components in the four groups of Liupao tea samples, which underwent different fermentation methods, exhibited variations in their categories. The content and percentage of different categories of aroma compounds in each group of samples were further analyzed, with the results presented in Figure 2a. It is evident that the different aroma compounds exhibited significant changes in their total amount and percentage. The relative total content of aroma components in the Cl group was higher in aldehydes, ketones, esters, and heterocyclic compounds, with values of 125.21 µg/kg, 104.75 µg/kg, 66.84 µg/kg, and 57.27 µg/kg, respectively, accounting for 21.32–26.08%, 18.96–21.24%, 11.91–13.77%, and 9.72–11.86% of the total content of aroma components. Similarly, the relative total content of aroma components in the CMl group was also higher in ketones, heterocyclic compounds, esters, and aldehydes, albeit with variations in content and proportion. The highest content and proportion were observed for ketones and heterocyclic compounds, with values of 78.98 µg/kg and 63.74 µg/kg, respectively, accounting for 19.71–22.31% and 15.41–18.41% of the total content of aroma components. The categories with higher average relative content of aroma components in the Dl group differed significantly from those in the Cl and CMl groups. The relative contents of alkenes and alcohols were 101.97 µg/kg and 81.66 µg/kg, respectively, accounting for 18.54–23.9% and 16.13–17.87% of the total aroma components. Ketones, alcohols, esters, and heterocyclic compounds were relatively higher in the DMl group, with values of 194.33 µg/kg, 181.68 µg/kg, 120.67 µg/kg, and 114.28 µg/kg, respectively, accounting for 23.3–23.94%, 23.13–21.05%, 14.55–14.77%, and 13.67–14.11% of the total content of aroma components. The total amount and classification of aroma components in the four groups of Liupao tea subjected to different fermentation methods were significantly different, as shown in Figure 2b,c. The total amount of aroma components in the four groups followed the order of DMl (822.97 ± 19.66 µg/kg) > Cl (523.08 ± 50.47 µg/kg) > Dl (480.53 ± 2.39 µg/kg) > CMl (375.55 ± 49.50 µg/kg). Significant differences were observed among the four groups in terms of the total amount of various aroma components (Figure 2d). The DMl group exhibited significantly higher levels of ketones and heterocyclic compounds compared to the other groups, with the total amount of aroma components in multiple categories being higher than in the other three groups. In addition to the high content of halogenated hydrocarbons and alkenes in the Dl and CMl groups, the total amount of other aroma components remained relatively low. While the DMl group had the highest total amount of aroma components and high levels of each type of aroma component, this alone could not fully explain its aroma quality characteristics. Therefore, further analysis of specific aroma components is necessary to identify the key characteristic aroma components contributing to its flavor quality.

### 3.4. Multivariate Statistical Analysis of Volatile Aroma Components

**Figure 3:**
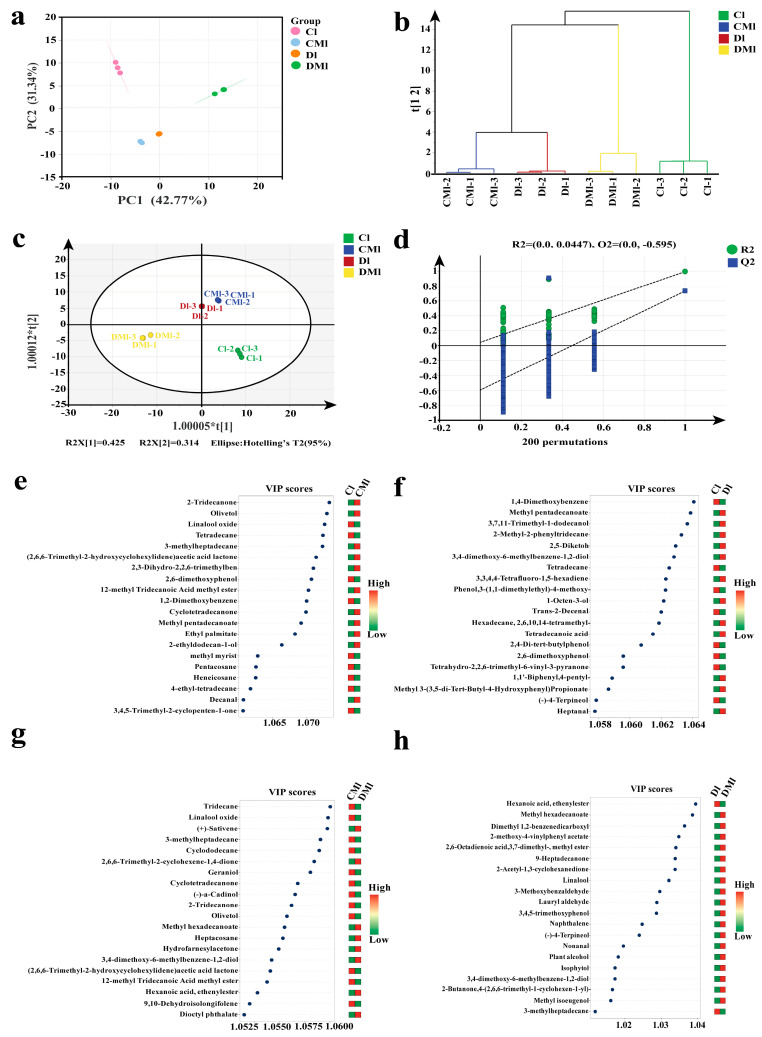
Scatter plot of PCA model scores of volatile compounds of tea samples. (**a**) Hierarchical cluster analysis (HCA) plot. (**b**) OPLS–DA model score of volatile compounds of tea samples. (**c**) Permutation test for validation of OPLS–DA model. (**d**) Differential compounds VIP value plot (Cl vs. CMl). (**e**) Differential compounds’ VIP value plot Cl vs. Dl). (**f**) Differential compounds’ VIP value plot (CMl vs. DMl). (**g**) Differential compounds’ VIP value plot (Dl vs. DMl). (**h**) Screening criterion for differential aroma compounds with VIP > 1, *p* < 0.05 and top 20 VIP ranking.

PCA is an unsupervised discriminant method, which can maximize the components of data variation, reduce data from a high dimension to a low dimension, and ensure orthogonality between each dimension. The reliability of the PCA model is strengthened when the PC cumulative ratio approaches 1. Following the detection of 156 aroma components, PCA dimensionality reduction and HCA were conducted (Figure 3a,b). The PCA results indicated clear differentiation among the four sample groups, with a PC1 variance contribution rate of 42.77%, a PC2 variance contribution rate of 31.34%, and a cumulative variance contribution rate of 74.11%, demonstrating a certain degree of reliability for the PCA model. HCA further supported the distinct clustering of the four sample groups, signifying significant differences in the aroma profiles of Liupao tea resulting from different fermentation methods. Specifically, the Cl group was clearly distinguishable from the CMl, Dl, and DMl groups, indicating differences in aroma composition between traditional cold fermentation and other fermentation methods. Additionally, the DMl and CMl/Dl groups were clearly distinguishable, highlighting significant differences in aroma profiles resulting from different fermentation methods used to process Liupao tea samples.

In order to further explore the differential aroma components of the four groups of samples, OPLS-DA analysis was performed on the 156 aroma components (Figure 3c). By using the 156 aroma components as the dependent variable and the different fermentation methods as the independent variables, effective differentiation of the four samples of Liupao tea with different fermentation methods could be achieved through OPLS-DA analysis. The screening conditions for differential aroma components were based on VIP > 1 and *p* < 0.05, as indicated by the OPLS-DA model. The model prediction index (Q2) in this analysis was 0.993, the independent variable fitting index (Rx2) was 0.941, and the dependent variable fitting index (Ry2) was 0.997. With R2 and Q2 exceeding 0.5, the model fitting result was deemed acceptable [36]. After 200 substitution tests, as shown in Figure 3d, the intersection point of the Q2 regression line with the vertical axis was less than 0, suggesting that the model validation was effective and that overfitting was not present. Therefore, the results are considered reliable for the differential analysis of the fermentation mode of the aroma in the four different groups of Liupao tea samples.

### 3.5. Screening and Analysis of Differential Aroma Compounds

Differential metabolite screening was conducted using criteria of VIP > 1 and *p* < 0.05, and the results are shown in Figure S2. A total of 37 up-regulated and 44 down-regulated aromas were identified in the Cl and CMl groups when compared to each other. Further analysis focused on the top 20 differential volatiles, ranked by VIP values, is depicted in Figure 3e, including compounds such as 2-tridecanone, linalool oxide, and olivetol. The results following the screening of the Cl and Dl groups were compared, revealing 41 up-regulated and 39 down-regulated aromas. The top 20 differential volatiles, characterized by VIP values, for both groups are shown in Figure 3f, which includes substances such as 1,4-dimethoxybenzene, methyl pentadecanoate, and 3,7,11-trimethyl-1-dodecanol. When evaluating the CMl group and DMl group, it was observed that there were 56 up-regulated and 30 down-regulated aroma components present. The differences in the top 20 VIP-ranked volatiles between these groups are shown in Figure 3g, which mainly includes compounds such as tridecane, linalool oxide, and (+)-sativene. Comparing the Dl group and the DMl group, 56 aroma components were up-regulated and 23 were down-regulated. Notably, the DMl group displayed a substantial increase in 18 up-regulated differential aroma components, including dimethyl 1,2-benzenedicarboxyl and 2-methoxy-4-vinylphenyl acetate (Figure 3h). The aroma components of the four different groups were quite different. These aroma components may potentially serve as marker compounds to differentiate between various fermentation methods of Liupao tea, although further analysis is required.

### 3.6. OAV Analysis of Differential Aroma Compounds

GC-Q-TOF-MS analysis was successful in characterizing and quantifying volatile aroma components. However, it was unable to specifically identify the characteristic aroma compounds responsible for the aroma profiles of the four sample groups. To address this limitation, we used the OAV of volatiles to identify the major volatiles present in the four samples subjected to different fermentation methods. By calculating the OAV of the potentially labeled aroma compounds, a total of 34 aroma compounds were identified (Table S3). These compounds encompass a range of sensory aromas, such as woody notes represented by (−)-4-terpineol, herbal notes represented by methyl salicylate, stale notes represented by 1,2-dimethoxybenzene, fruity notes represented by benzyl alcohol, and floral notes represented by linalool. In addition, the analysis revealed the presence of other aroma compounds with distinct profiles, such as acetophenone with an almond aroma and 1-octen-3-ol with a mushroom aroma.

**Figure 4:**
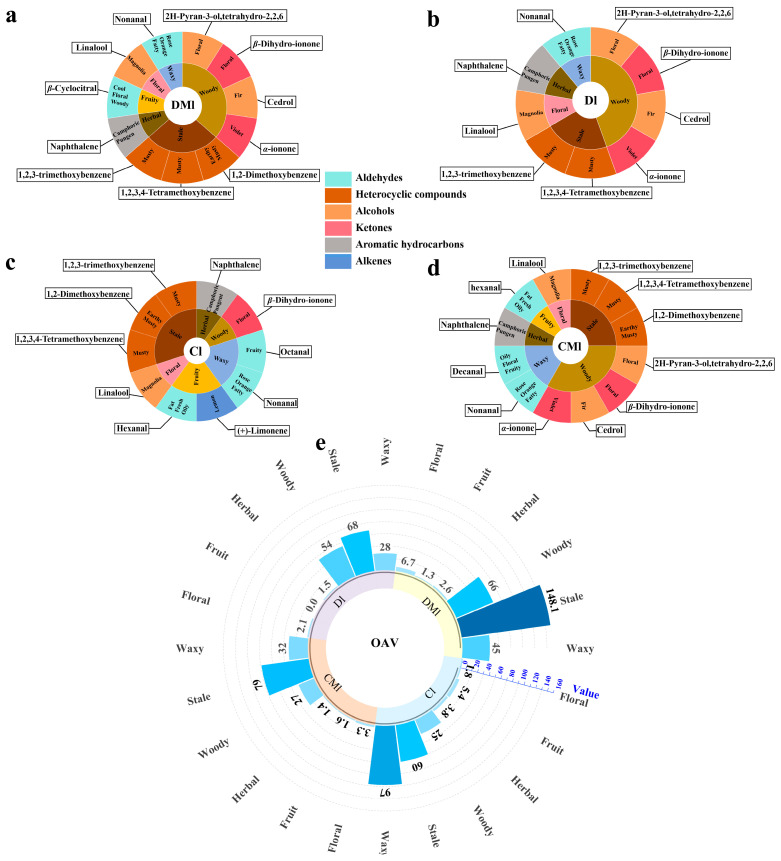
Flavor wheel of key volatile compounds in the four types of Liupao tea samples. DMl group (**a**); Dl group (**b**); Cl group (**c**); CMl group (**d**); cumulative quantitative bar chart of OAV (**e**).

The key aroma compounds with OAV > 1 in the four groups of samples were screened to create the flavor wheel. The aroma types of Liupao tea were mainly dominated by stale and woody aromas; however, the specific aroma components differed in type and quantity (Figure 4a–d). The OAVs of these characteristic aroma compounds of different aroma types in each group were further quantified (Figure 4e). Differences were observed in the OAV cumulative quantization values of the different aroma types across the four groups of Liupao tea samples. The OAV cumulative quantization values for the DM1 group, specifically for stale and woody aromas, were significantly higher compared to the other groups. The OAV cumulative quantization value for the stale aroma feature was notably high, reaching 148.1. Stale aroma is a distinctive characteristic of high-quality Liupao tea and is positively correlated with its overall quality [5]. In general, the DM1 group exhibited prominent stale and woody aroma characteristics compared to the other groups using different fermentation methods; moreover, in the OAV, accumulation values for other aroma types were also higher, indicating superior overall aroma quality.

**Figure 5:**
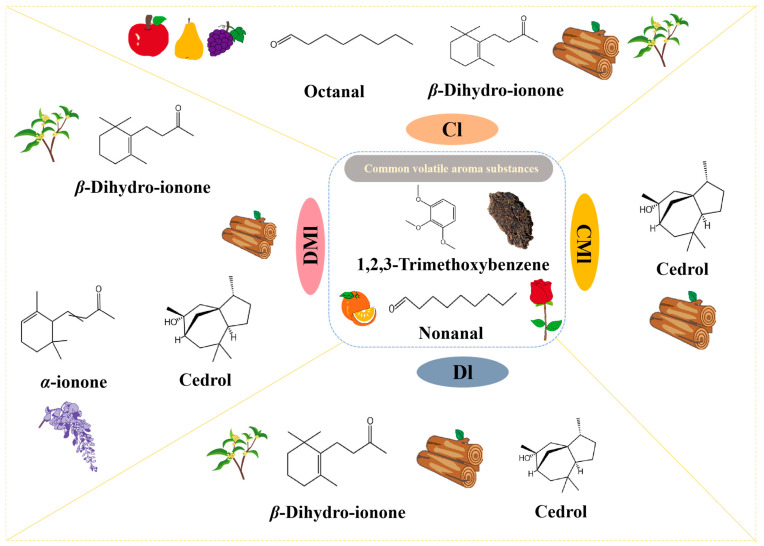
The key volatile compounds in the four types of samples (OAV > 10).

The greater the OAV of an aroma compound, the greater its influence on the sample aroma. When the OAV > 10, the compound is considered to have a significant contribution to the overall aroma of the tea [37]. A screening of key characteristic aroma compounds was further carried out with OAV > 10 as the screening condition, as shown in Figure 5. The analysis revealed that the characteristic aroma compounds shared among the four fermentation methods used in the production of Liupao tea are 1,2,3-trimethoxybenzene with a stale aroma and nonanal with floral and fruit aromas. In the Cl group, octanal provided a strong fruity aroma, which made an important contribution to the fruity characteristics of this group. The CMl group was characterized by cedrol with a woody fragrance, while the Dl group contained cedrol as well as β-Dihydro-ionone, offering both floral and woody notes. It is worth noting that the aroma profile of the DMl group exhibited greater diversity compared to the other three groups. This observation may be attributed to the inoculation of raw materials with *M. purpureus* during the initial steaming process. The presence of *M. purpureus* appears to enhance the abundance of aromas in the DMl group, contributing to its unique profile [38].

### 3.7. Metabolic Pathways of Key Characteristic Aroma Compounds

**Figure 6:**
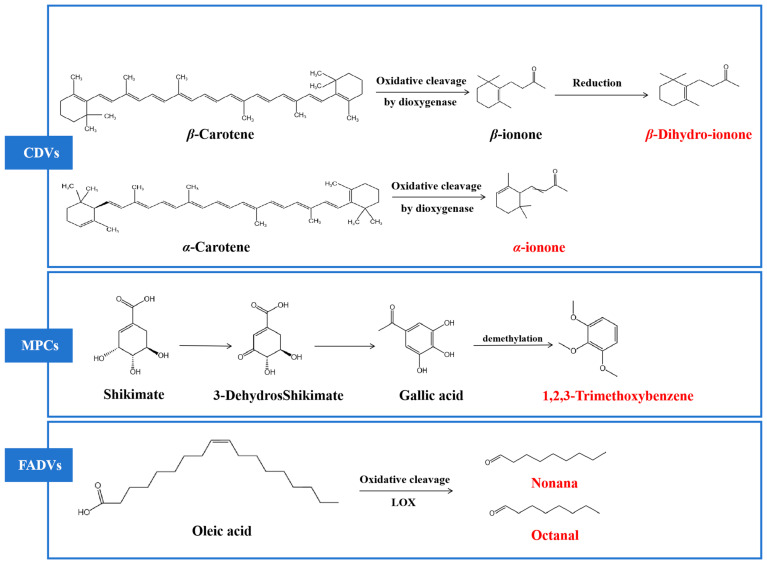
Pathways for the formation of key characteristic aromatic compounds.

The changes in aroma characteristics during tea processing are affected by various factors. The six key characteristic aroma components identified in this study can be divided into fatty acid derivatives (FADVs), carotenoid-derived volatiles (CDVs), and methoxybenzenes (MPCs), as shown in Figure 6. FADVs can produce some short-chain C6–C9 fatty aldehydes through the action of lipoxygenase (LOX) and hydroperoxide lyase (HPL) as a result of microbial activity [39]. Nonanal and octanal, as FADVs, have floral and fruity properties and are generated by the oxidative degradation of oleic acid [40]. CDVs are derived from different carotenoid cleavage dioxygenases (CCDs), including β-carotene, α-carotene, phytoene, lutein and lycopene, during processes such as enzymatic oxidative degradation, photooxidation, autooxidation, or thermal degradation in fermentation and drying [41]. The key characteristic aromas in this study include two CDVs: α-ionone, which is mainly produced by the oxidative degradation of α-carotene, and dihydro β-ionone, which is a secondary oxidation product derived from β-carotene as well as a biotransformation product of β-ionone [42]. In addition, GA is methylated under the action of microbial enzymes to produce MPCs, such as 1,2,3-trimethoxybenzene and other fragrance compounds [43]. Cedrol, a sesquiterpene alcohol known for its woody aroma, has been identified as a key aroma compound in Liupao tea [44]. Previous research has shown a significant increase in cedrol during the early stages of fermentation of Fuzhuan brick tea, stabilizing towards the end of the process. At present, the metabolic pathway of cedar alcohol is not clear, and it is speculated that it may be related to high-temperature steam treatment or microbial activity [45,46]. The fermentation of Liupao tea caused significant changes in its flavor profile, influenced not by a single factor but by the combined effects of microbial metabolism, enzymatic oxidation, and moist heat. In the future, the key microbial flora involved in the fermentation process and their impact on the quality and flavor development of Liupao tea will be further explored.

## 4. Conclusions

The results of sensory evaluation indicated that fermentation by *M. purpureus* improved the quality of Liupao tea. When following traditional technology, the inoculation of *M. purpureus* fermentation resulted in a sample with a more reddish-brown appearance and infusion color, a sweeter and more mellow taste, and a more abundant aroma. Among the four Liupao teas with different fermentation methods, the DMl group exhibited the best sensory quality. Using HS-SPME-GC-Q-TOF/MS, a total of 156 volatile aroma substances across 13 categories were identified. E-nose detection results revealed distinct odor fingerprints for Liupao tea under various fermentation methods, with the sensors W1S (methyl group), W1W (inorganic sulfide), and W2S (alcohols and ketones) showing larger response contributions. PCA and OPLS-DA analyses demonstrated a significant separation of volatile components among the four groups of Liupao tea with different fermentation methods. Through differential aroma compounds screening based on VIP > 1 and *p* < 0.05, potential key aroma compounds were identified. These results, combined with OAV analysis and aroma flavor wheel construction, indicated that the Cl, CMl, Dl, and DMl groups contained 10, 12, 9, and 11 key odorants, respectively. By further screening for characteristic aroma compounds with OAV > 10, it was observed that 1,2,3-trimethoxybenzene with stale aroma and nonanal with floral and fruity aromas were shared by all fermentation modes of Liupao tea. In the DMl group, which had the highest total aroma amount, key characteristic aroma compounds included 1,2,3-trimethoxybenzene, nonanal, and cedrol. The OAV of 1,2,3-trimethoxybenzene was significantly higher in the DMl group, contributing the most to its characteristic flavor. These findings can provide guidance for the development of Liupao tea products and the optimization of processes, providing a comprehensive understanding of Liupao tea. In the future, GC-O-MS, aroma recombination experiments and omission tests can be used to further determine the actual aroma contribution of the key aroma components of Liupao tea fermented by different fermentation methods, and to further study the synergistic effect of each aroma component.

## Figures and Tables

**Table 1:** Sensory evaluation of the different fermentation methods of Liupao tea.

Sample	Shape	Taste	Fragrance	Soup Color	Leaf Base	Score A
Cl	Brownish-brown, tight, comparatively	Rich and full-bodied with a sweet aftertaste	Stale, herbal, cool, fruity	Deep red and bright	Brown, soft, and shiny	90.1
CMl	Red-brown, tight, comparatively	Sweet and mellow, Thick and smooth	Rich, stale, woody, and fruity	Brighter red intensity	Reddish-brown, soft, and shiny	90.45
Dl	Brownish-brown, nearly even and orderly	Rich and full-bodied with a bitter aftertaste	Stale with floral and fruit	Red and thick	Brown, soft, and shiny	90.15
DMl	Red-brown, nearly even and orderly	Sweet and mellow with a sweet aftertaste	Rich and stale with woody, floral, and fruity scents	Red, thicker and brighter	Reddish-brown, soft, and shiny	93

Score A: Appearance × 20%, beverage color × 15%, aroma × 25%, taste × 30%, leaf base × 10%, and two sensory reviews’ results and calculation of average. (Cl represents the finished product of Liupao tea processed by cold fermentation, while Dl represents the finished product of Liupao tea processed by hot fermentation. CMl and DMl represent the finished products of Liupao tea processed by cold and hot fermentation, respectively, combined with *M. purpureus* inoculation).

## Data Availability

The original contributions presented in the study are included in the article/Supplementary Materials, further inquiries can be directed to the corresponding authors.

## Supplementary Materials

The following supporting information can be downloaded at https://www.mdpi.com/article/10.3390/foods13162595/s1, Table S1: Sensors used in PEN3 system and their main applications. Table S2: Mean relative contents of aroma components of Liupao tea with different fermentation methods. Table S3. Odor activity values (OAVs) of key volatile substances and their aroma characteristics. Figure S1. Production of Monascus purpureus liquid. Figure S2. Processing of tea samples. Figure S3. (A) Differential Aroma Compounds Sankey Diagram (Cl vs. CMl); (B) Differential Aroma Compounds Sankey Diagram (Cl vs. Dl); (C) Differential Aroma Compounds Sankey Diagram (CMl vs. DMl); (D) Differential Aroma Compounds Sankey Diagram (Dl vs. DMl), The screening criteria were VIP > 1, *p* < 0.05.
