# Supplementary material for: Effects of Different Fermentation Methods on Flavor Quality of Liupao Tea Using GC-Q-TOF-MS and Electronic Nose Analyses
Source: Foods. 2024 Aug 19;13(16):2595. doi: 10.3390/foods13162595 (PMC11353607; doi:10.3390/foods13162595)
Supplement: Supplementary file 1 [file foods-13-02595-s001.zip › foods-3119456-supplementary.pdf]

Supplementary Table S1. Sensors used in PEN3 system and their main applications.

Sensor number	Sensor name	Main applications
R1	W1C	Sensitive to aromatic compounds
R2	W5S	Broad sensitivity and very sensitive to nitrogen oxides
R3	W3C	Sensitive to ammonia and aromatic compounds
R4	W6S	Mainly sensitive to hydrogen
R5	W5C	Sensitive to alkenes and aromatic compounds
R6	W1S	Sensitive to methane
R7	W1W	Sensitive to sulfides
R8	W2S	Sensitive to alcohol
R9	W2W	Sensitive to aromatic compounds and organic sulfides
R10	W3S	Mainly sensitive to alkenes

Supplementary Table S2. Mean relative contents of aroma components of Liupao tea with different fermentation methods

NO.	Retention Time (min)	class	Name	CAS	Formula	Absolute Content (μg/Kg)			
						Cl	CMl	Dl	DMl
1	8.90	alcohols	Benzyl alcohol	100-51-6	C7H8O	2.27±0.33	0.97±0.14	-	-
2	11.72	alcohols	Linalool	78-70-6	C10H18O	6.99±1.99	12.43±4.8	7.99±2.08	25.5±3.02
3	14.72	alcohols	2H-Pyran-3-ol,tetrahydro-2,2,6	14049-11-7	C10H18O2	4.19±0.27	6.86±0.88	10.01±0.88	24.45±2.5
4	15.07	alcohols	(-)-4-Terpineol	20126-76-5	C10H18O	4.18±0.13	0.92±0.03	1.96±0.22	4.33±0.51
5	21.92	alcohols	2-(2,6,6-trimethylcyclohexen-1-yl)ethanol	472-65-1	C11H20O	1.2±0.01	-	1.56±0.06	1.09±0.04
6	22.58	alcohols	Linalool oxide	1365-19-1	C10H18O3	1.45±0.01	0.77±0.06	-	-

7	25.20	alcohols	Cyclododecanol	1724-39-6	$C_{12}H_{24}O$	4.82±0.13	1.18±0.3	-	-
8	28.60	alcohols	1-Tridecanol	112-70-9	$C_{13}H_{28}O$	1.49±0.03	-	-	-
9	34.76	alcohols	(-)-a-Cadinol	481-34-5	$C_{15}H_{26}O$	3.57±2.07	0.98±0.1	1.77±0.11	-
10	44.88	alcohols	Isophytol	505-32-8	$C_{20}H_{40}O$	3.79±0.52	4.63±0.07	2.61±0.46	5.39±0.56
11	45.91	alcohols	2-Hexyl-1-n-octanol	19780-79-1	$C_{14}H_{30}O$	0.9±0.25	-	-	-
12	46.80	alcohols	Plant alcohol	150-86-7	$C_{20}H_{40}O$	1.14±0.09	0.51±0.27	0.54±0.09	1.07±0.1
13	15.66	alcohols	alpha-Terpineol	98-55-5	$C_{10}H_{18}O$	2.91±0.1	5.06±0.76	-	-
14	6.92	alcohols	1-Octen-3-ol;	3391-86-4	$C_8H_{16}O$	-	3.73±0.07	2.75±0.21	6.31±0.27
15	18.56	alcohols	Geraniol	106-24-1	$C_{10}H_{18}O$	-	2.79±0.25	-	-
16	36.20	alcohols	2-ethyldodecan-1-ol	19780-33-7	$C_{14}H_{30}O$	-	0.38±0.06	-	-
17	48.91	alcohols	3,7,11-Trimethyl-1-dodecanol;	6750-34-1	$C_{15}H_{32}O$	-	0.58±0.01	1.32±0.09	1.37±0.13
18	49.06	alcohols	hexadecanol	36653-82-4	$C_{16}H_{34}O$	-	4.1±0.13	3.17±0.83	14.47±3.42
19	32.70	alcohols	cedrol	77-53-2	$C_{15}H_{26}O$	-	7.6±1.37	17.26±0.03	15.03±4.32
20	17.38	alcohols	Cyclohexanol,5-methyl-2-(1-methylethenyl)-	89-79-2	$C_{10}H_{18}O$	-	-	0.75±0.25	-
21	24.21	alcohols	3,4-dimethoxy-6-methylbenzene-1,2-diol	54826-79-8	$C_9H_{12}O_4$	-	-	0.93±0.07	1.78±0.21
22	41.33	alcohols	3,7,11,15-tetramethyl-2-hexadecen-1-ol	102608-53-7	$C_{20}H_{40}O$	-	-	29.02±3.43	52.64±1.42

23	59.01	alcohols	2,3,4-trimethylpentan-1-ol	6570-88-3	C8H18O	-	-	0.02±0	-
24	11.25	alcohols	(Z)-linalool oxide (furanoid)	5989-33-3	C10H18O2	-	-	-	26.23±0.65
25	13.38	alcohols	(-)-trans-pinocarveol	547-61-5	C10H16O	-	-	-	0.56±0.18
26	16.25	alcohols	Borneol	507-70-0	C10H18O	-	-	-	1.45±0.21
27	24.85	alkanes	Tetradecane	629-59-4	C14H30	17.37±1.31	3.89±0.07	-	3.3±0.39
28	40.30	alkanes	Hexadecane	544-76-3	C16H34	4.88±0.15	5.24±0.88	6.68±0.39	6.82±2.46
29	52.53	alkanes	Heneicosane;	629-94-7	C21H44	1.92±0.3	-	0.93±0.33	2.23±0.5
30	54.91	alkanes	Heptacosane	593-49-7	C27H56	0.39±0.1	-	-	1.19±0.13
31	55.34	alkanes	Tetracosane	646-31-1	C24H50	2.1±0	-	-	-
32	62.51	alkanes	Pentacosane	629-99-2	C25H52	0.94±0.14	-	-	-
33	36.48	alkanes	Heptadecane	629-78-7	C17H36	0.73±0.12	-	2.26±0.12	4.4±0.59
34	38.30	alkanes	4-ethyl-tetradecane	55045-14-2	C16H34	2.13±0.34	-	-	-
35	6.51	alkanes	propan-2-ylcyclobutane	872-56-0	C7H14	0.7±0.25	-	-	-
36	19.57	alkanes	1,2-Epoxyhexadecane	7320-37-8	C16H32O	4.98±0.11	-	1.37±0.04	0.57±0
37	51.56	alkanes	2,2-Bis(4-hydroxyphenyl)propane	80-05-7	C15H16O2	0.26±0.11	-	0.23±0.03	0.48±0.02
38	20.58	alkanes	Tridecane	629-50-5	C13H28	-	1.15±0.09	-	-

39	27.84	alkanes	Cyclododecane	294-62-2	C12H24	-	1.6±0.13	1.58±0.25	-
40	27.91	alkanes	Acenaphthene	83-32-9	C12H10	-	0.19±0.01	-	-
41	36.69	alkanes	2,6,10-Trimethyldodecane	3891-98-3	C15H32	-	1.25±0.07	1.29±0	-
42	38.97	alkanes	3-methylheptadecane	6418-44-6	C18H38	-	0.9±0.08	1.36±0.38	-
43	39.98	alkanes	n-Octadecane	593-45-3	C18H38	-	2.3±0.54	2.05±0.24	5.25±0.07
44	40.31	alkanes	Hexadecane, 2,6,10,14-tetramethyl-	96990-16-8	C20H42	-	0.74±0.18	1.1±0.08	2.12±0.02
45	28.91	alkanes	Pentadecane;	629-62-9	C15H32	-	11.69±15.33	-	-
46	42.93	alkanes	2-Methyl-2-phenyltridecane	27854-41-7	C20H34	-	-	1.64±0.12	-
47	42.95	alkanes	1,2-Epoxyoctadecane	7390-81-0	C18H36O	-	-	3.19±0.02	-
48	7.58	alkanes	4-bromocyclohexene	3540-84-9	C6H9Br	-	-	-	1.44±0.6
49	16.17	alkanes	2,6,11-Trimethyldodecane	31295-56-4	C15H32	-	-	-	3.66±0.28
50	36.20	alkanes	germacrane	645-10-3	C15H30	-	-	-	2.4±3.12
51	45.63	alkanes	1-Iodooctadecane	629-93-6	C18H37I	-	-	-	4.02±2.96
52	46.97	alkanes	1-methyl-2-nonyl-1-propan-2-ylcyclopropane	41977-40-6	C16H32	-	-	-	0.18±0
53	7.13	ketones	6-Methyl-5-hepten-2-one	110-93-0	C8H14O	2.1±0.47	-	2.83±0.4	5.96±1.61
54	9.18	ketones	3-Octen-2-one	1669-44-9	C8H14O	1.63±0.45	-	-	-

55	10.22	ketones	Acetophenone	98-86-2	C8H8O	26.79±0.36	-	-	-
56	12.02	ketones	Tetrahydro-2,2,6-trimethyl-6-vinyl-3-pyranone	33933-72-1	C10H16O2	11.39±1.14	46.2±10.93	-	92.03±0.92
57	20.30	ketones	2-Undecanone	112-12-9	C11H22O	2.28±0.11	1.43±0.21	-	6.59±2.38
58	24.12	ketones	2-Acetyl-1,3-cyclohexanedione	4056-73-9	C8H10O3	3.15±0.13	1.86±0.19	2.19±0.12	3.51±0.23
59	26.38	ketones	β -Dihydro-ionone	17283-81-7	C13H22O	2.27±0.66	0.77±0.13	1.11±0.14	1.79±0.09
60	27.01	ketones	Geranylacetone	3796-70-1	C13H22O	10.64±1.55	3.67±0.28	7.59±0.3	15.02±0.63
61	27.65	ketones	2,5-Di-tert-butyl-1,4-benzoquinone	2460-77-7	C14H20O2	0.48±0.2	0.27±0.06	0.38±0.04	-
62	33.67	ketones	Diphenylmethanone	119-61-9	C13H10O	0.11±0	-	0.9±0.01	1.79±0.32
63	41.59	ketones	Phytone	502-69-2	C18H36O	42.35±1.92	9.09±1.02	25.88±6.6	49.51±0.24
64	53.36	ketones	3-benzylcyclobutan-1-one	55262-02-7	C11H12O	1.56±0.06	-	-	-
65	10.42	ketones	2-Cyclohexen-1-one,5-(1-hydroxy-1-methylethyl)-2-methyl-, (5S)-	60593-11-5	C10H16O2	-	5.66±1.27	5.32±0.13	8.12±0.42
66	13.61	ketones	2,6,6-Trimethyl-2-cyclohexene-1,4-dione	1125-21-9	C9H12O2	-	0.71±0.13	1.89±0.02	2.29±0.05
67	15.96	ketones	Cyclohexanone, 2-methyl-5-(1-methylethenyl)-, trans-	6909-25-7	C10H16O	-	0.79±0.21	-	-
68	25.93	ketones	α-ionone	127-41-3	C13H20O	-	1.03±0.07	2.1±0.04	4.8±0.88
69	28.30	ketones	β-ionone	79-77-6	C13H20O	-	5.53±0.16	-	-

70	28.75	ketones	2-Tridecanone;	593-08-8	C13H26O	-	0.19±0.02	-	-
71	42.64	ketones	Cyclotetradecanone	3603-99-4	C14H28	-	1.79±0.18	-	-
72	9.24	ketones	5-ethenyl-5-methyloxolan-2-one	1073-11-6	C7H10O2	-	-	1.12±0.05	-
73	18.07	ketones	2,5-Diketoh	110-13-4	C6H10O2	-	-	1.97±0.15	-
74	36.42	ketones	2-Pentadecanone	2345-28-0	C15H30O	-	-	0.88±0.1	-
75	22.31	ketones	9-Heptadecanone	540-08-9	C17H34O	-	-	-	1.93±0.4
76	43.93	ketones	hydrofarnesylacetone	1117-52-8	C18H30O	-	-	-	0.99±0.12
77	6.63	esters	4-(4-Aminophenyl)butanoic acid	20637-09-6	C11H15NO2	2.82±1.09	-	-	-
78	7.03	esters	Hexanoic acid, ethenyl ester	3050-69-9	C8H14O2	6.57±1.43	-	9.74±0.64	4.89±0.6
79	15.83	esters	Methyl salicylate	119-36-8	C8H8O3	4.85±0.09	-	-	-
80	21.61	esters	2,6-Octadienoic acid,3,7-dimethyl-, methyl ester	2349-14-6	C11H18O2	3.36±1.16	2.24±0.21	-	3.06±0.63
81	27.05	esters	Dimethyl 1,2-benzenedicarboxyl	131-11-3	C10H10O4	6.72±1.1	7.14±0.53	9.46±0.65	24.55±2.87
82	37.39	esters	methyl myrist	124-10-7	C15H30O2	1.69±0.02	0.63±0.18	0.81±0.05	1±0.06
83	43.79	esters	Diisobutyl phthalate	84-69-5	C16H22O4	3.22±0.55	6.15±3.37	6.18±1.25	16.68±3.94
84	44.23	esters	Methyl hexadecanoate	112-39-0	C17H34O2	29.77±2.63	22.25±1.93	31.01±2.01	44.98±1.58
85	49.41	esters	Methyl linoleate	112-63-0	C19H34O2	5.7±1.75	4.5±1.59	3.56±0.24	3.5±0

86	50.42	esters	methyl stearate	112-61- 8	C19H38 O2	1.4±0.03	0.94±0.22	1.19±0.25	1.79±0.0 6
87	12.56	esters	3-hexenyl ester, (Z)-He xanoic acid	31501-1 1-8	C12H22 O2	-	6.1±1.64	-	10.77±1. 34
88	15.86	esters	methyl o-acetylsalicyla te;	580-02- 9	C10H10 O4	-	0.53±0.21	-	-
89	29.88	esters	(2,6,6-Trimethyl-2-hyd roxycyclohexylidene)acetic acid lactone	17092-9 2-1	C11H16 O2	-	2.4±0.29	-	-
90	39.87	esters	12-methyl Tridecanoic Acid methyl ester	5129-58 -8	C15H30 O2	-	0.96±0.11	-	-
91	40.89	esters	Methyl pentadecanoate	7132-64 -1	C16H32 O2	-	0.49±0.06	0.65±0.04	0.59±0.0 2
92	46.38	esters	Ethyl palmitate	628-97- 7	C18H36 O2	-	0.73±0.08	0.26±0.09	0.48±0.0 3
93	46.49	esters	Hexadecanoicacid, 14- methyl-, methyl ester	2490-49 -5	C18H36 O2	-	1.03±0.02	0.68±0.1	-
94	17.07	esters	Methyl 2-methylvalerat e	2177-77 -7	C7H14O 2	-	-	0.89±0.01	2.03±0.0 4
95	44.70	esters	Methyl 3-(3,5-di-Tert- Butyl-4-Hydroxyphenyl)Pro pionate	6386-38 -5	C18H28 O3	-	-	0.09±0.01	-
96	51.33	esters	9-(o-Propylphenyl)non anoic acid methyl ester	17670-8 6-9	C19H30 O2	-	-	0.24±0	0.45±0.0 2
97	21.09	esters	2-methoxy-4-vinylphen yl acetate	46316-1 5-8	C11H12 O3	-	-	-	0.63±0.1 3
98	26.48	esters	6-Ketotetradecanoic ac id methyl ester	24243-1 7-2	C15H28 O3	-	-	-	2.14±0.0 2
99	47.36	esters	Isopropyl palmitate	142-91- 6	C19H38 O2	-	-	-	2.93±3.5 1
100	40.12	esters	Octyl salicylate	118-60- 5	C15H22 O3	0.73±0.03	-	-	-

101	64.71	esters	Dioctyl phthalate	117-84-0	$C_{24}H_{38}O_4$	-	-	-	0.19±0.02
102	2.23	aldehydes	hexanal	66-25-1	$C_6H_{12}O$	11.86±1.4	7.21±1.47	-	-
103	7.72	aldehydes	Octanal	124-13-0	$C_8H_{16}O$	15.35±0.09	-	-	-
104	11.91	aldehydes	Nonanal	124-19-6	$C_9H_{18}O$	74.95±27.2	25.33±1.44	28.14±3.9	45.46±1.81
105	17.00	aldehydes	β-Cyclocitral	432-25-7	$C_{10}H_{16}O$	2.61±0.1	1.74±0.16	-	3.88±0.62
106	18.85	aldehydes	trans-2-Decenal	3913-81-3	$C_{10}H_{18}O$	3.13±0.26	-	-	-
107	39.25	aldehydes	1-hexadecanal	629-80-1	$C_{16}H_{32}O$	17.31±3.04	6.21±1.96	32.59±2.53	2.87±0.3
108	16.08	aldehydes	2,3-Dihydro-2,2,6-trimethylben	116-26-7	$C_{10}H_{14}O$	-	0.66±0.06	0.87±0.17	1.51±0.48
109	16.41	aldehydes	Decanal;	112-31-2	$C_{10}H_{20}O$	-	12.55±2.24	-	-
110	28.19	aldehydes	4-(1,1-dimethylethyl)-Benzenepropanal	18127-01-0	$C_{13}H_{18}O$	-	0.11±0.02	-	-
111	4.44	aldehydes	Heptanal	111-71-7	$C_7H_{14}O$	-	-	2.97±0.32	-
112	18.45	aldehydes	3-Methoxybenzaldehyde	591-31-1	$C_8H_8O_2$	-	-	0.99±0.02	4.4±0.75
113	23.27	aldehydes	(E)-2-Octenal;	2548-87-0	$C_8H_{14}O$	-	-	1.62±0.02	1.57±0.04
114	25.19	aldehydes	Lauryl aldehyde	112-54-9	$C_{12}H_{24}O$	-	-	-	1.7±0.37
115	31.84	aldehydes	pentadecanal	2765-11-9	$C_{15}H_{30}O$	-	-	-	0.77±0.01
116	46.59	aldehydes	(Z)-13-Octadecenal	58594-45-9	$C_{18}H_{34}O$	-	-	-	0.95±0

117	8.72	alkenes	(+)-Limonene	5989-27-5	C10H16	27.98±1.37	-	-	-
118	22.14	alkenes	3,4,5-Trimethyl-2-cyclopenten-1-one	55683-21-1	C8H12O	2.31±0.22	0.57±0.2	0.81±0.12	1.09±0.07
119	23.46	alkenes	3,4-Dimethoxystyrene;	6380-23-0	C10H12O2	2.08±0.37	6.36±2.67	1.54±0.04	3.33±0.25
120	29.25	alkenes	9,10-Dehydroisolongifolene	67530-11-4	C15H22	0.49±0.17	0.17±0.02	0.46±0.04	-
121	13.98	alkenes	4-Methoxystyrene	637-69-4	C9H10O	-	1.53±0.08	-	-
122	4.51	alkenes	3,3,4,4-Tetrafluoro-1,5-hexadiene	1763-21-9	C6H6F4	-	-	2.49±0.21	-
123	24.31	alkenes	(+)-Cyclosativene	22469-52-9	C15H24	-	-	96.67±13.28	2.77±0.06
124	1.18	alkenes	1,2-Difluoroethylene	689-99-6	C2F2	-	-	-	5.13±0.32
125	8.80	alkenes	limonene	138-86-3	C10H16	-	-	-	6.23±2.65
126	21.65	alkenes	(+)-Sativene	3650-28-0	C15H24	-	-	-	0.74±0.06
127	18.10	phenols	2,6-dimethoxyphenol	91-10-1	C8H10O3	21.05±1.93	3.4±0.19	1.88±0.21	8.58±0.81
128	25.05	phenols	Methyl eugenol	93-15-2	C11H14O2	0.7±0.2	-	-	-
129	29.41	phenols	2,4-Di-tert-butylphenol;	96-76-4	C14H22O	2.34±0.21	2.05±0.62	-	-
130	31.17	phenols	4-Propoxyphenol	18979-50-5	C9H12O2	4.31±1.29	2.52±0.74	1.52±0.07	2.61±0.12
131	47.58	phenols	2,6-di-t-amyl-4-methylphenol	56103-67-4	C17H28O	0.25±0.09	-	-	-
132	29.85	phenols	Olivetol	500-66-3	C11H16O2	-	7.24±0.78	-	-

133	29.45	phenols	tert-Butylhydroquinone	1948-33-0	C10H14O2	-	-	0.72±0.12	-
134	29.40	phenols	2,5-Di-tert-butylphenol	5875-45-6	C14H22O	-	-	-	4.8±2.58
135	33.17	phenols	3,4,5-trimethoxyphenol	642-71-7	C9H12O4	-	-	-	0.44±0.09
136	8.56	aromatic hydrocarbons	1,2,3,5-tetramethylbenzene	527-53-7	C10H14	2.54±0.1	-	-	-
137	20.05	aromatic hydrocarbons	2-Methylnaphthalene	91-57-6	C11H10	3.02±0.03	0.51±0.03	0.66±0.03	-
138	39.11	aromatic hydrocarbons	2,2',5,5'-Tetramethylbiphenyl	3075-84-1	C16H18	0.28±0.02	0.27±0.04	0.17±0.01	0.52±0.02
139	15.45	aromatic hydrocarbons	Naphthalene	91-20-3	C10H8	3.81±0.82	1.38±0.1	1.45±0.28	2.62±0.03
140	34.23	aromatic hydrocarbons	1,1'-Biphenyl,4-pentyl-;	7116-96-3	C17H20	-	0.87±0.03	0.95±0.1	2.53±0.07
141	7.58	aromatic hydrocarbons	2-Propylfuran	4229-91-8	C7H10O	-	0.71±0.14	-	-
142	20.15	aromatic hydrocarbons	Indole	120-72-9	C8H7N	-	-	1.66±0.08	-
143	13.77	heterocyclic compounds	1,2-Dimethoxybenzene	91-16-7	C8H10O2	15.5±0.53	6.6±1.09	-	5.34±0.06

144	14.47	heterocyclic compounds	1,4-Dimethoxybenzene	150-78-7	C8H10O2	1.01±0.06	-	-	-
145	21.21	heterocyclic compounds	1,2,3-trimethoxybenzene	634-36-6	C9H12O3	35.29±7.93	54.12±12.37	48.85±4.53	103.87±0.96
146	26.80	heterocyclic compounds	1,2,3,4-Tetramethoxybenzene	21450-56-6	C10H14O4	5.47±2.02	2.78±0.18	1.53±0.37	5.07±0.39
147	17.90	heterocyclic compounds	3,4-Dimethoxytoluene	494-99-5	C9H12O2	-	0.25±0	-	-
148	38.66	acids	Tetradecanoic acid	544-63-8	C14H28O2	0.3±0	0.67±0.23	0.7±0.04	2.28±0.1
149	50.53	acids	Linoleic acid	60-33-3	C18H32O2	1.87±0.4	-	-	-
150	45.46	acids	Hexadecanoic acid	57-10-3	C16H32O2	18.39±1.76	11.27±0.75	24.76±1.32	61.35±1.71
151	42.00	acids	Pentadecanoic acid	1002-84-2	C15H30O2	-	-	0.61±0.34	-
152	21.42	ethers	Phenol,3-(1,1-dimethylethyl)-4-methoxy-	88-32-4	C11H16O2	1.52±0.12	-	-	-
153	27.20	ethers	Methyl isoeugenol	93-16-3	C11H14O2	0.48±0.13	0.24±0.03	1.01±0.06	2.37±0.34
154	1.76	amines	3,5-Dihydroxybenzamide	3147-62-4	C7H7NO3	-	-	-	0.61±0.19
155	16.78	amines	N-Formanilide	103-70-8	C7H7NO	-	-	-	2.83±0.07
156	35.13	alkanes	1-Chlorooctadecane	3386-33-2	C18H37Cl	-	0.68±0.04	0.65±0.04	-

Supplementary Table S1. Odor activity values (OAVs) of key volatile substances and their aroma characteristics.

NO.	CAS	Compounds	OT(μg/L)	Sample				Aroma Type
				Cl	CMl	Dl	DMl	
1	14049-11-7	2H-Pyran-3-ol,tetrahydro-2,2,6	6.00	0.70	1.14	1.67	4.08	Woody, Floral
2	20126-76-5	(-)-4-Terpineol	330.00	0.01	__	0.01	0.01	Spicy, Herbal
3	17283-81-7	β -Dihydro-ionone	0.09	25.22	8.55	12.3 7	19.87	Woody, floral
4	91-10-1	2,6-dimethoxyphenol	29.00	0.73	0.12	0.06	0.30	Woody, herbal

5	77-53-2	cedrol	0.50	__	15.21	34.51	30.07	Woody, Fir
6	127-41-3	α-ionone	0.40	__	2.57	5.24	11.99	Woody, violet
7	116-26-7	2,3-Dihydro-2,2,6-trimethylben	3.00	__	0.22	0.29	0.50	Woody, Herb, swee, spicy
8	98-55-5	alpha-Terpineol	300.00	0.01	0.02	__	__	Anise, Mint, Floral
9	119-36-8	Methyl salicylate	40.00	0.12	__	__	__	Mint, Peppermint, Anise, herbal, Almond
10	93-15-2	Methyl eugenol	68.00	0.01	__	__	__	Anise, mint, spicy, herbal
11	91-57-6	2-Methylnaphthalene	10.00	0.30	0.05	0.07	__	Tar, pungent
12	91-20-3	Naphthalene	1.00	3.81	1.38	1.45	2.62	Tar, camphoric and greasy odor, pungent
13	91-16-7	1,2-Dimethoxybenzene	3.17	4.89	2.08	__	1.68	Stale, Earthy, musty
14	634-36-6	1,2,3-trimethoxybenzene	0.75	47.06	72.16	65.13	138.50	Stale, musty
15	21450-56-6	1,2,3,4-Tetramethoxybenzene	0.64	8.54	4.34	2.39	7.92	Stale, musty
17	100-51-6	Benzyl alcohol	100.00	0.02	0.01	__	__	Fruity, apple

18	110-93-0	6-Methyl-5-hepten-2-one	50.00	0.04	__	0.06	0.12	Fruity, lemon
19	66-25-1	hexanal	4.50	2.63	1.60	__	__	Fruity Fat, Fresh, Green, Oil
20	628-97-7	Ethyl palmitate	2.00	__	0.36	0.13	0.24	Fruity, cream, waxy
21	112-54-9	Lauryl aldehyde	2.00	__	__	__	0.85	Fruity
22	5989-27-5	(+)-Limonene	10.00	2.80	__	__	__	Fruity, Lemon, sweet
16	120-72-9	Indole	140.00	__	__	0.01	__	Floral, Jasmine, lilac
23	1365-19-1	Linalool oxide	6.00	0.24	0.13	__	__	Floral, cool, Woody
24	3796-70-1	Geranylacetone	60.00	0.18	0.06	0.13	0.25	Floral, rose, magnolia
25	629-80-1	1-hexadecanal	75.00	0.23	0.08	0.43	0.04	Floral, Waxy
26	78-70-6	Linalool	3.80	1.84	3.27	2.10	6.71	Floral, magnolia
27	36653-82-4	hexadecanol	750.00	__	0.01	__	0.02	Flower, Wax
28	106-24-1	Geraniol	75.00	__	0.04	__	__	Floral, rose
29	98-86-2	Acetophenone	2000.00	0.01	__	__	__	Nutty, Floral
30	124-13-0	Octanal	0.70	21.93	__	__	__	Pungent, Fruity
31	124-19-6	Nonanal	1.00	74.95	25.33	28.1	45.46	Waxy, rose-like, fresh, oran

						4		ge-like, fatty
32	432-25-7	β-Cyclocitral	3.00	0.87	0.58	—	1.29	Cool, fruity
33	3391-86-4	1-Octen-3-ol	10.00	—	0.37	0.28	0.63	Mushroom, earthy, green, oily
34	112-31-2	Decanal;	1.97	—	6.37	—	—	Waxy, oil, sweet, wax, floral, fruity

Threshold query using https://www.vcf-online. and Compilation of odor threshold values in air, water and other media.

Odor description found in the literature with database (http://www.leffingwell.com).

“ —” No identified.

Figure Captions

Supplementary Figure S1. Production of *Monascus purpureus* liquid

Supplementary Figure S2. Processing of tea samples

Supplementary Figure S3. (A)Differential Aroma Compounds Sankey Diagram(Cl vs CMl);(B)Differential Aroma Compounds Sankey Diagram(Cl vs Dl);(C) Differential

Aroma Compounds Sankey Diagram(CMl vs DMl);(D)Differential Aroma Compounds Sankey Diagram(Dl vs DMl),The screening criteria were VIP > 1,P < 0.05.

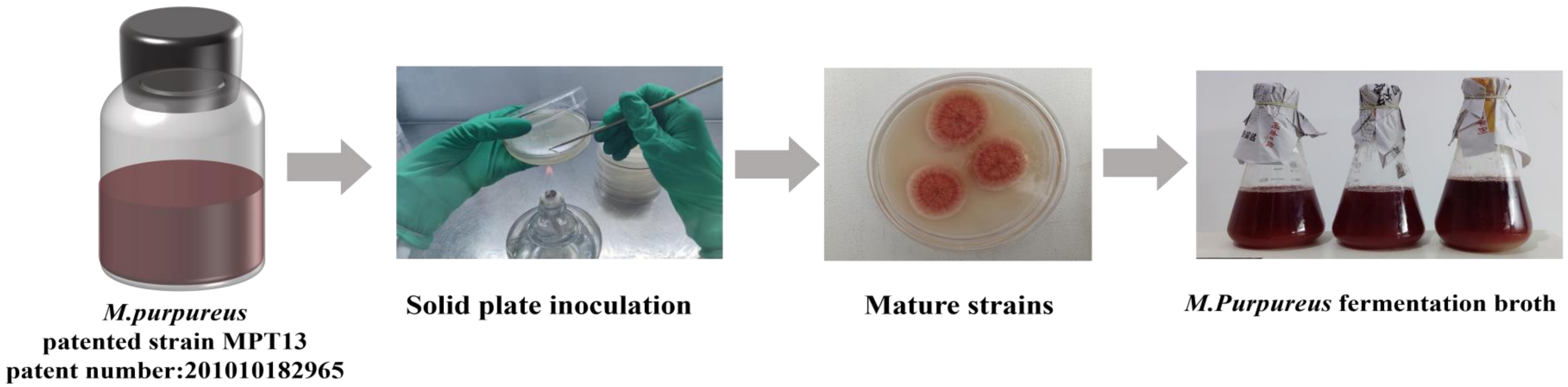

Supplementary Figure S1.

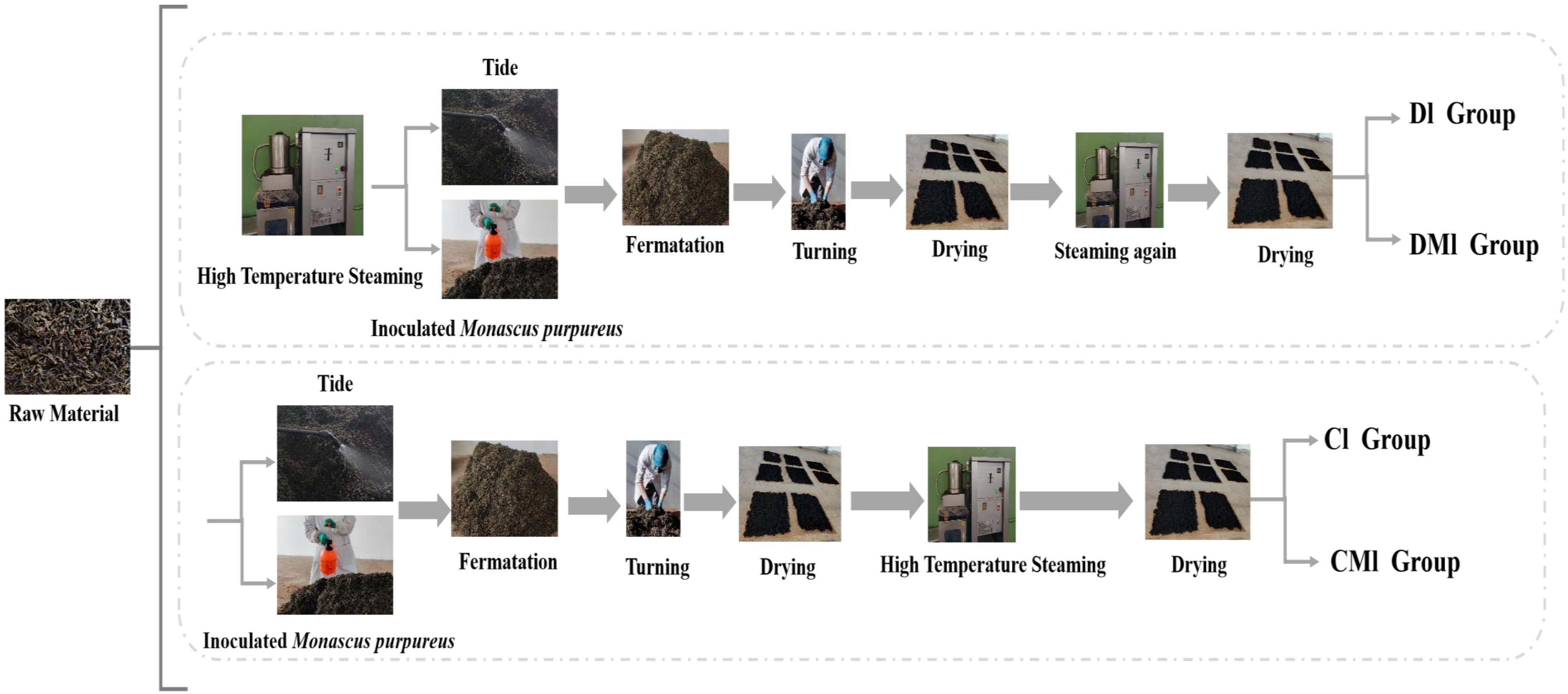

Supplementary Figure S2.

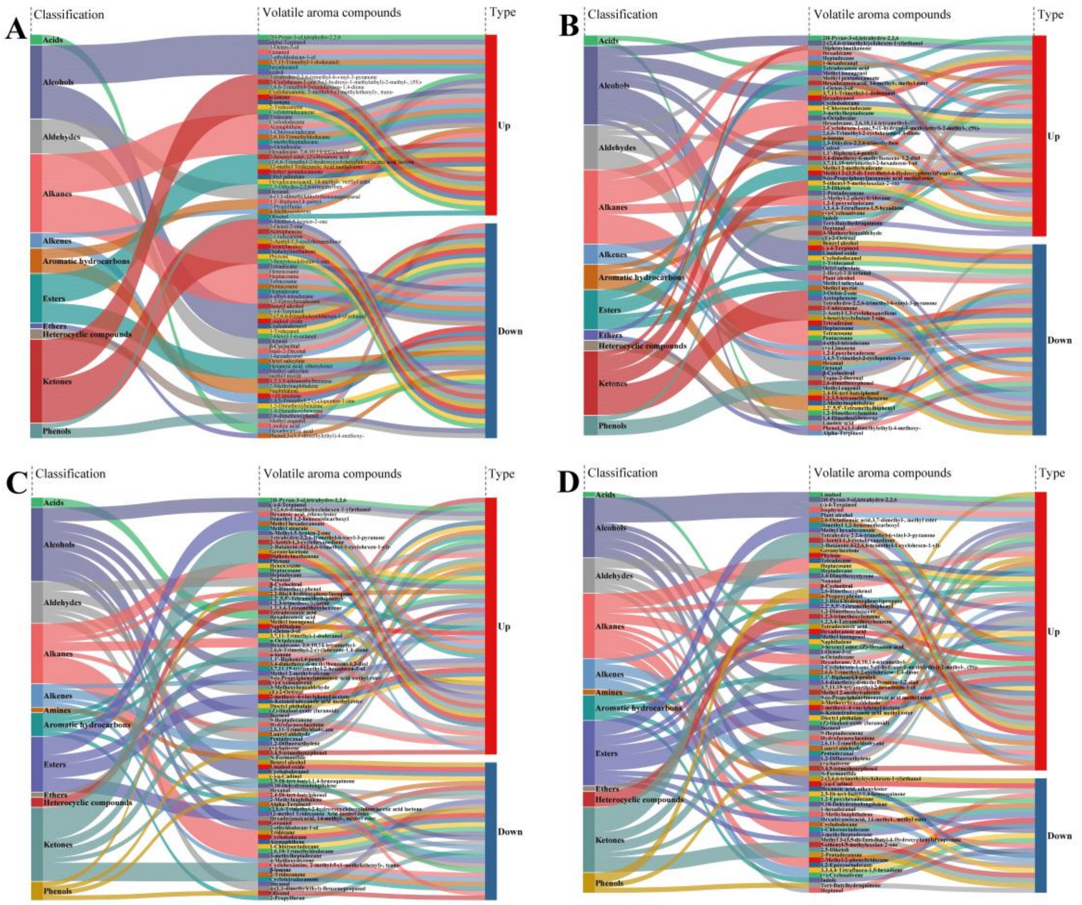

Supplementary Figure S3.
